# Characteristics of COVID-19 Patients Based on the Results of Nucleic Acid and Specific Antibodies and the Clinical Relevance of Antibody Levels

**DOI:** 10.3389/fmolb.2020.605862

**Published:** 2021-01-12

**Authors:** Hao Chen, Rundong Qin, Zhifeng Huang, Li He, Wenting Luo, Peiyan Zheng, Huimin Huang, Hui Wang, Baoqing Sun

**Affiliations:** ^1^State Key Laboratory of Respiratory Disease, Department of Allergy and Clinical Immunology, Guangzhou Institute of Respiratory Health, First Affiliated Hospital of Guangzhou Medical University, Guangzhou, China; ^2^Department of Medical Laboratory, The Central Hospital of Wuhan, Tongji Medical College, Huazhong University of Science and Technology, Wuhan, China

**Keywords:** COVID-19, SARS-CoV-2, nucleic acid, antibody, clinical outcomes

## Abstract

Combination of nucleic acid and specific antibody testing is often required in the diagnosis of COVID-19, but whether patients with different nucleic acid and antibody results have different laboratory parameters, severities and clinical outcomes, has not yet been comprehensively investigated. Thus, according to different groups of nucleic acid and antibody results, we aimed to investigate the differences in demographic characteristics, and laboratory parameters among the different groups and predict their clinical outcomes. In our study, nasopharyngeal swab nucleic acids and antibodies were detected by reverse-transcription polymerase chain reaction and chemiluminescence, respectively. Patients with confirmed COVID-19 with different severities, were divided into the PCR^+^Ab^+^, PCR^+^Ab^−^, and PCR^−^Ab^+^ groups. Demographic characteristics, symptoms, comorbidities, laboratory parameters, and clinical outcomes were compared among the three groups. The correlation of antibodies with laboratory parameters and clinical outcomes was also explored, and antibodies were used to predict the timing of nucleic acid conversion. We found that a total of 364 COVID-19 patients were included in the final analysis. Of these, a total of 184, 37, and 143 patients were assigned to the PCR^+^Ab^+^, PCR^+^Ab^−^, and PCR^−^Ab^+^ groups, respectively. Compared to patients in the PCR^+^Ab^−^ or PCR^−^ Ab^+^ groups, patients in the PCR^+^Ab^+^ group presented worse symptoms, more comorbidities, more laboratory abnormalities, and worse clinical outcomes (*P* < 0.05). In addition, the levels of IgG, IgM, and IgA were all significantly correlated with the days of hospitalization, days of PCR turning negative, and multiple laboratory parameters (*P* < 0.05). Meanwhile, combined IgM, IgA, and IgG predicted the days of PCR turning negative within 1 week. The best performance was achieved when the cut-off values of IgM, IgG, and IgA were 3.2, 1.8 and 0.5, respectively, with a sensitivity of 73% and specificity of 82%. In conclusion, COVID-19 patients who were both positive for nucleic acids and antibodies presented with worse clinical features, laboratory abnormalities, and clinical outcomes. The three specific antibodies were positively correlated with clinical outcomes and most laboratory parameters. Furthermore, antibody levels can predict the time of nucleic acid conversion.

## Introduction

The rapid spread of severe acute respiratory syndrome coronavirus 2 (SARS-CoV-2) has led to more than 28 million individuals being infected and has caused more than 0.9 million fatal cases, leading to tremendous human and economic losses worldwide, the impact of this disease is expected to continue (World Health Organization, 2020a). The identification of clinical characteristics in patients with COVID-19 of different types is crucial to decrease the mortality rate and achieve favorable clinical outcomes. Currently, most COVID-19 patients are being tested for nucleic acids and serological antibodies before admission, and this even precede the severity assessment. Therefore, the characteristics of COVID-19 patients grouped by the results of polymerase chain reaction (PCR) and serological antibodies may play an important role in the development of COVID-19. Researchers have focused on the clinical characteristics, risk factors, and laboratory parameters of COVID-19 patients with different severities (Guan et al., 2020), but whether patients with different nucleic acid and antibody results have different laboratory parameters, severities and clinical outcomes has not yet been comprehensively investigated.

Currently, PCR-based SARS-CoV-2 RNA detection from respiratory samples, as the gold standard, and serological antibody tests, as the supplemental methods, provide direct and indirect evidence of COVID-19 infection. Thus, this has been widely used in point-of-care for COVID-19 (World Health Organization, 2020b). Several factors can affect the results of PCR and serological antibody tests (Liu R. et al., 2020; Zhang W. et al., 2020). For example, the adequacy of the specimen collection technique, time from exposure, and specimen source are known to affect the results of PCR, while different testing methods, variable antigen or antibody preparation, and timing of the detection are reported to be associated with the accuracy of serological antibody tests. Most importantly, the intensity of the immune response is a common factor that affects both PCR and serological antibodies test by the abilities of virus clearance and production of antibodies, respectively (Liu et al., 2020b). An impaired immune system is the predominant feature of COVID-19 infection, as evidenced by an instant upregulated inflammatory response leading to the subsequent inflammatory storm (Tang et al., 2020).

In the current study, we hypothesized that the different results obtained on PCR and serological antibody tests of patients with COVID-19 are representative of different conditions of immune system response against the virus, which presents various clinical features and laboratory abnormalities. Using a retrospective cohort to screen patients with confirmed COVID-19 who were hospitalized for <7 days since symptom onset, we divided the included patients into the following three subgroups: (1) patients with both positive PCR and serological antibodies test results, (2) patients with positive PCR and negative serological antibody test results, and (3) patients with negative PCR and positive serological antibody test results. The differences in clinical features, outcomes, and laboratory parameters were compared among the three groups, and the clinical relevance of antibody levels was further investigated.

## Materials and Methods

### Patients

Adult COVID-19 patients hospitalized at Wuhan Central Hospital from February 10 to March 26, 2020, were included. The diagnosis met the Guidelines of the Diagnosis and Treatment of New Coronavirus Pneumonia (version 7) published by the National Health Commission of China (National Health Commission of China, 2020): (1) suspected case, and (2) evidence of etiology or serology. According to the status of the patient during the time of admission, the disease was clinically classified as mild, moderate, severe, and critical. Mild disease was characterized by mild symptoms, with no manifestations of pneumonia on computed tomography (CT) imaging; Moderate disease was characterized by fever, respiratory symptoms and other associated symptoms (i.e., fatigue, dyspnea, diarrhea, muscle soreness), and the manifestation of viral pneumonia on CT; and severe disease met at least one of the following additional conditions: (1) shortness of breath with respiratory rate ≥30 breaths/min, (2) oxygen saturation at rest ≤93%, and (3) oxygenation index ≤300 mmHg. Those with critical disease had at least one of these additional conditions: (1) respiratory failure requiring mechanical ventilation, (2) shock, and (3) failure of other organs, possibly requiring admission to the intensive care unit (ICU). Regarding the collection of patients' epidemiological data (i.e., the onset of symptoms, complications, and imaging data), to avoid the influence of medications, the initial laboratory test results on admission, including hematological, biochemical, coagulation, inflammatory, and immunological parameters, record on admission in patients with different severities, days of hospitalization, and days of PCR turning negative. This study was approved by the Ethics Committee of Wuhan Central Hospital Medical (Research Ethics No. 1, 2020). Considering the infectivity of COVID-19, informed consent was not required to be signed.

### Nucleic Acid and Antibody Detection

Nasopharyngeal swabs and serum samples from all included COVID-19 patients were collected. The 2019-nCoV nucleic acid detection kit provided by Shanghai Zhijiang Biotechnology Co., Ltd. and a real-time fluorescence quantitative PCR instrument were used for detection. Cycle thresholds <37 and >40 were considered positive and negative, respectively. After the serum was inactivated in a water bath at 56°C for 30 min, SARS-CoV-2-specific IgM, IgA, and IgG were detected by chemiluminescence immunoassay (CLIA) using the reagents provided by Tianjin Bioscience Co., Ltd. A single-probe CLIA was conducted on an Axceed 260 automatic CLIA analyzer (Bioscience, Tianjin, China), and the specific antibodies were directed against the receptor-binding domain of the spike protein (S protein). The cut-off values of IgA, IgM, and IgG were 67219.8, 53292.5, and 73400.9, respectively. The relative luminescence value (RLV) ≥1.0 was positive for specific IgA, IgM, and IgG.

### Study Protocol

All patients were admitted to the hospital within 1 week after symptom onset. To avoid false- negative results in nucleic acid testing, patients with negative initial PCR test result were assessed at least two times, and patients with positive PCR test result the first time were assessed at least four times. The result was defined as positive if it appeared positive once, and it was considered negative only if it was negative in every single test. Because several studies have proposed that specific IgA is equally important in SARS-CoV-2 infection (Guo et al., 2020; Jaaskelainen et al., 2020), in addition to the commonly performed IgM and IgG antibody detection, we also included the specific IgA of SARS-CoV-2. Three antibodies were tested on admission; whenever one of the antibodies was positive, it was defined as positive; the result was considered as negative when all the three antibodies were negative. Days of PCR turning negative were calculated using the time of the first positive PCR as the starting time and the time of the first negative PCR as the cut-off time. Patient discharge criteria were as follows: absence of fever for at least 3 days, substantial improvement in both chest CT and respiratory symptoms, and a negative viral RNA obtained from two nasopharyngeal swab samples at least 24 h apart.

### Statistical Analyses

Normally distributed continuous data are expressed as mean and standard deviation, and non-normally distributed continuous data are expressed as median and quartile intervals. The χ^2^ test or Fisher's exact probability test was used to compare qualitative data. The Mann–Whitney *U*-test was used for independent sample comparison between the two groups of non-parametric data. The Kruskal–Wallis H test was used for comparison between multiple groups. For statistical purposes, we grouped severe and critical patients into a category. *P* < 0.05 was considered statistically significant. The Statistical Package for the Social Sciences version 23.0 (International Business Machines Corporation, Armonk, NY, USA) and GraphPad Prism version 8.0.1 (©1995–2020; GraphPad Software, LLC, San Diego, CA, USA) were used for data analyses. We used Gephi (version 0.9.2; GitHub, Inc., San Francisco, CA, USA) to construct the network graphics of the clinical outcomes and laboratory parameters.

## Results

### Patient Characteristics

A total of 411 confirmed adult COVID-19 patients were recruited, of whom four died and 43 patients with missing data on many laboratory parameter were excluded. Therefore, 364 patients were included in the final analysis (Table 1). Of these patients, 184, 37, and 143 patients were assigned to the PCR^+^Ab (antibody)^+^, PCR^+^Ab^−^, and PCR-Ab^+^ groups, respectively. The positive rate of nucleic acid detection was 61%, and the median ages of the three groups were 62, 53, and 56, respectively. The age of patients in the PCR^+^Ab^+^ group was significantly higher than that in the PCR-Ab+ group (*P* < 0.001). According to the severity of COVID-19 status at the time of admission, a total of 29, 298, and 37 patients were grouped into the severe/critical, moderate, and mild categories, respectively. Comparing the PCR^+^Ab^+^ and PCR^+^Ab^−^ groups, more number of patients with severe/critical and moderate disease and fewer with mild disease were observed in the former than the latter group (*P* = 0.002). Higher positive chest CT findings were also observed in the PCR^+^Ab^+^ group (*P* = 0.033). Fever was the main symptom on admission in all three groups, affecting 64.67, 37.83, and 58.04% of the subjects in each group, respectively, followed by cough, fatigue, and dyspnea. Patients in the PCR^+^Ab^+^ group had a significantly higher incidence of major symptoms than those in the PCR^+^Ab^−^ and PCR^−^Ab^+^ groups (*P* < 0.001 and *P* = 0.001, respectively). In terms of comorbidities, the proportions of patients with hypertension and diabetes in the PCR^+^Ab^+^ were 40.76 and 18.47%, respectively, significantly higher than that of PCR^−^Ab^+^ group (*P* < 0.001).

**Table 1:** Characteristics, disease severity, symptoms, and comorbidity of included patients with COVID-19.

	**Overall *N* = 364**	**PCR^+^Ab^+^*N* = 184**	**PCR^+^Ab^−^*N* = 37**	**PCR^−^Ab^+^*N* = 143**	**Comparison (*P*-value)**	
					**PCR^+^Ab^+^ vs. PCR^+^Ab^−^**	**PCR^+^Ab^+^ vs. PCR^−^Ab^+^**
**Characteristics**						
Age (years)	59.00 (46.00;69.00)	62.00 (49.00;70.00)	53.00 (38.00;84.00)	56.00 (43.00;63.00)	0.814	**<0.001**
Male (*n*, %)	155 (42.58)	82 (44.57)	16 (43.24)	57 (39.86)	0.315	0.431
Smoker (*n*, %)	32 (8.79)	15 (8.15)	3 (8.11)	14 (9.79)	0.911	0.715
**Severity**						
Severe/critical (*n*, %)	29 (7.97)	17 (9.23)	3 (8.18)	9 (6.29)	**0.002**	0.619
Moderate (*n*, %)	298 (81.87)	152 (82.61)	23 (62.16)	123 (86.01)		
Mild (*n*, %)	37 (10.16)	15 (8.15)	11 (29.72)	11 (7.69)		
**HR chest-CT**	349 (95.88)	177 (96.20)	32 (86.49)	140 (97.90)	**0.033**	0.817
**Symptoms**						
Fever (*n*, %)	216 (59.34)	119 (64.67)	14 (37.83)	83 (58.04)	**<0.001**	**0.001**
Cough (*n*, %)	196 (53.84)	113 (61.41)	13 (35.14)	70 (48.95)		
Fatigue (*n*, %)	166 (45.60)	98 (53.26)	9 (24.32)	59 (41.26)		
Dyspnea (*n*, %)	108 (29.67)	63 (34.24)	7 (18.91)	38 (26.57)		
Diarrhea (*n*, %)	30 (8.24)	15 (8.15)	2 (5.41)	13 (9.09)		
Sore throat (*n*, %)	17 (4.67)	9 (4.89)	2 (5.41)	6 (4.20)		
Muscle soreness (*n*, %)	6 (1.64)	1 (0.54)	2 (5.41)	3 (2.10)		
**Comorbidity**						
Hypertension (*n*, %)	131 (35.99)	75 (40.76)	15 (40.54)	41 (28.67)	0.084	**<0.001**
Diabetes (*n*, %)	60 (16.48)	34 (18.47)	3 (8.11)	23 (16.08)		
Heart related disease (*n*, %)	53 (14.56)	32 (17.39)	6 (16.22)	15 (10.49)		
Hyperlipidemia (*n*, %)	14 (3.85)	9 (4.89)	2 (5.41)	3 (2.10)		
Malignancy (*n*, %)	34 (9.34)	26 (14.13)	2 (5.41)	6 (4.19)		
Chronic gastritis (*n*, %)	14 (3.85)	11 (5.94)	0 (0.00)	3 (2.10)		
COPD (*n*, %)	17 (4.67)	8 (4.34)	1 (2.71)	8 (5.59)		

*P < 0.05 are shown in bold*.

*COVID-19, Coronavirus Disease 2019; CT, Computed Tomography; COPD, Chronic Obstructive Pulmonary Disease; PCR, Polymerase Chain Reaction; Ab, Antibody; +, Positive; –, Negative*.

### Laboratory Parameters in the Three Groups

Hematological, biochemical, coagulation, inflammatory, and immunological parameters are considered to be related to the severity and prognosis of COVID-19 patients, so these laboratory parameters were checked in all the patients we included. The results of the analyses of the laboratory parameters are shown in Table 2. As regards hematological parameters, comparing the PCR^+^Ab^+^ and PCR^−^Ab^+^ groups, patients in the former group had significantly higher *neutrophil* (Neu) *percentage* (*P* < 0.001), and lower *red blood cell (RBC)* counts, *lymphocyte (Lym) percentage* and *basophil (Bas) percentage* than those in the latter group (*P* = 0.001, *P* < 0.001, and *P* = 0.005, respectively). Among the three groups, patients in the PCR^+^Ab^+^ group had significantly lower *eosinophil (Eos)* counts than the patients in the other two groups (*P* = 0.012 and *P* < 0.001, respectively). Regarding biochemical parameters, comparing the PCR^+^Ab^+^ and PCR^−^Ab^+^ groups, patients in the former group had significantly higher levels of *aspartate aminotransferase (AST), hydroxybutyrate dehydrogenase (HBDH)*, and *lactate dehydrogenase (LDH)* (*P* = 0.004, *P* < 0.001, *P* < 0.001, respectively) and lower levels of *total protein, potassium, sodium* than those in the latter group (*P* = 0.028, *P* = 0.013 and *P* = 0.008, respectively). Comparing the PCR^+^Ab^+^ and PCR^+^Ab^−^ groups, patients in the former group had significantly lower level of *direct bilirubin* (*P* = 0.035) and higher levels of *glucose* (*P* = 0.032) and *gamma-glutamyl transpeptidase (GGT)* (*P* = 0.030). Among the three groups, patients in the PCR^+^Ab^+^ group had significantly lower levels of *albumin* (*P* = 0.009 and *P* < 0.001, respectively) and *phosphorus* (*P* = 0.026 and *P* < 0.001, respectively) than the other two groups. As regards coagulation parameters, comparing the PCR^+^Ab^+^ and PCR^−^Ab^+^ groups, patients in the former group had significantly higher *prothrombin time (PT)* and *prothrombin time ratio (PTR)* (both *P* = 0.034) and lower *prothrombin active* (PTA) (*P* = 0.029). Among the three groups, patients in the PCR^+^Ab^+^ group had significantly higher *D-dimer* levels than those in the other two groups (*P* = 0.014 and *P* < 0.001, respectively). In terms of inflammatory parameters, comparing the PCR^+^Ab^+^ and PCR^−^Ab^+^ groups, patients in the former group had significantly higher levels of *C-reactive protein (CRP)* and *procalcitonin (PCT)* than those in the latter group (*P* < 0.001 and *P* = 0.041, respectively). Regarding immunological parameters, among the three groups, patients in the PCR^+^Ab^+^ group had significantly higher levels of *Krebs von den Lungen-6 (KL-6)* (*P* = 0.001 and *P* = 0.024, respectively) and specific IgA (*P* < 0.001 and *P* = 0.012, respectively) than the other two groups.

**Table 2:** Laboratory parameters of COVID-19 patients with different result of PCR and antibodies test.

**Laboratory parameters**	**PCR^+^Ab^+^*N* = 221**	**PCR^+^Ab^−^*N* = 37**	**PCR^−^Ab^+^*N* = 143**	**Comparison (*P-*value)**	
				**PCR^+^Ab^+^ vs. PCR^+^Ab^−^*p*-value**	**PCR^+^Ab^+^ vs. PCR^−^Ab^+^*p*-value**
**Hematological parameters**					
WBC	4.83 (2.01; 7.16)	4.59 (2.00; 6.93)	5.00 (2.27; 7.05)	0.972	0.640
RBC	4.19 (3.74; 4.53)	4.41 (3.69; 4.74)	4.30 (4.04; 4.68)	0.445	**0.001**
Neu (%)	65.90 (57.40; 76.20)	63.00 (58.55; 72.70)	59.90 (53.35; 68.90)	0.583	**<0.001**
Lym (%)	24.60 (15.90; 31.95)	26.10 (17.80; 31.70)	29.80 (21.58; 35.30)	0.424	**<0.001**
Eos (%)	0.90 (0.20; 1.90)	1.40 (0.55; 2.35)	1.80 (0.98; 3.10)	**0.012**	**<0.001**
Bas (%)	0.30 (0.20; 0.50)	0.30 (0.20; 0.55)	0.40 (0.20; 0.60)	0.143	**0.005**
Mono (%)	7.10 (5.56; 9.00)	6.50 (4.91; 9.00)	6.80 (5.70; 8.13)	0.749	0.072
PLT	209.00 (158.00; 263.50)	194.00 (150.00; 254.00)	208.50 (170.75; 250.25)	0.238	0.589
Hb	126.00 (116.00; 137.00)	132.00 (115.50; 145.00)	128.00 (119.75; 138.25)	0.778	0.264
**Biochemical parameters**					
Albumin	38.00 (34.20; 42.00)	41.90 (37.30; 43.90)	40.70 (36.65; 44.10)	**0.009**	**<0.001**
Globulin	27.00 (24.20; 30.80)	26.40 (23.00; 29.30)	26.60 (23.70; 30.00)	0.053	0.101
ALT	18.70 (12.45; 34.45)	15.40 (9.85; 26.65)	20.90 (15.03; 34.08)	0.069	0.698
AST	20.00 (14.73; 26.08)	17.90 (14.40; 32.25)	18.00 (14.27; 23.78)	0.110	**0.004**
CK	63.00 (45.00; 95.00)	79.00 (47.00; 110.10)	57.00 (39.00; 82.00)	0.085	0.526
Creatinine	65.00 (53.35; 85.75)	68.00 (53.05; 85.40)	60.70 (49.50; 79.95)	0.339	0.732
Total bilirubin	10.40 (7.50; 14.65)	11.10 (7.80; 16.00)	10.05 (7.57; 14.00)	0.102	0.966
Direct bilirubin	3.40 (2.00; 4.90)	4.40 (2.50; 5.45)	2.90 (1.40; 4.20)	**0.035**	0.097
Indirect bilirubin	7.00 (5.50; 9.90)	8.20 (5.85; 10.20)	7.35 (5.38; 9.90)	0.286	0.339
Glucose	5.16 (4.43; 5.99)	4.69 (4.48; 6.23)	4.98 (4.60; 5.94)	**0.032**	0.105
GGT	20.20 (14.00; 35.50)	16.00 (11.20; 25.75)	24.20 (14.70; 49.78)	**0.030**	0.201
HBDH	143.00 (119.00; 175.00)	133.00 (116.00; 162.00)	116.50 (103.00; 148.25)	0.277	**<0.001**
LDH	177.50 (144.00; 219.00)	160.00 (142.00; 192.00)	152.00 (134.00; 192.25)	0.296	**<0.001**
Total protein	67.00 (63.53; 71.78)	68.00 (63.10; 71.65)	68.60 (62.50; 71.83)	0.627	**0.028**
Cl	104.50 (102.23; 106.40)	104.20 (101.75; 106.10)	104.40 (102.50; 106.25)	0.940	0.193
K	4.20 (3.85; 4,43)	4.24 (4.03; 4.45)	4.26 (3.98; 4.49)	0.093	**0.013**
Na	139.70 (138.08; 141.23)	140.00 (138.10; 141.90)	140.75 (138.43; 142.80)	0.418	**0.008**
P	1.04 (0.90; 1.18)	1.12 (0.99; 1.24)	1.15 (0.99; 1.28)	**0.026**	**<0.001**
**Coagulation parameters**					
PTA	105.00 (90.73; 118.80)	101.00 (92.50; 122.70)	107.20 (94.50; 118.80)	0.398	**0.029**
PT	11.50 (11.00; 12.18)	11.55 (10.85; 12.25)	11.30 (10.90; 11.80)	0.521	**0.034**
APTT	28.40 (25.83; 32.10)	27.70 (25.30; 31.70)	27.75 (24.80; 30.08)	0.776	0.234
PTR	0.99 (0.95; 1.05)	0.99 (0.93; 1.06)	0.97 (0.94; 1.02)	0.421	**0.034**
TT	16.40 (15.70; 17.48)	16.20 (14.90; 16.80)	16.40 (15.90; 17.38)	0.227	0.563
D-dimer	0.72 (0.28; 1.90)	0.37 (0.12; 0.98)	0.43 (0.18; 1.24)	**0.014**	**<0.001**
**Inflammatory parameters**					
CRP	0.54 (0.11; 2.82)	0.37 (0.11; 1.37)	0.15 (0.07; 1.45)	0.189	**<0.001**
PCT	0.05 (0.04; 0.08)	0.05 (0.04; 0.07)	0.04 (0.03; 0.06)	0.767	**0.041**
**Immunological parameters**					
CD4^+^T%	43.18 (36.27; 47.75)	44.62 (40.83; 50.05)	43.71 (36.40; 49.47)	0.188	0.438
CD8^+^T%	24.44 (19.67; 30.93)	21.63 (17.87; 28.31)	24.94 (20.47; 30.41)	0.095	0.859
CD4^+^T/CD8^+^T	1.69 (1.31; 2.24)	1.95 (1.47; 2.77)	1.74 (1.22; 2.40)	0.078	0.683
KL-6	270.00 (184.50; 401.50)	170.00 (127.00; 320.00)	253.00 (182.00; 375.00)	**0.001**	**0.024**
IgM	16.28 (3.59; 45.12)	0.10 (0.06; 0.31)	20.70 (7.07; 47.43)	**<0.001**	0.555
IgG	54.97 (10.87; 95.47)	0.23 (0.14; 0.54)	68.72 (36.70; 104.01)	**<0.001**	0.548
IgA	10.01 (2.71; 26.91)	0.23 (0.17; 0.26)	9.61 (3.40; 22.80)	**<0.001**	**0.012**

*P < 0.05 are shown in bold*.

*COVID-19, Coronavirus Disease 2019; PCR, Polymerase Chain Reaction; Ab, Antibody; +, Positive; –, Negative; WBC, White Blood Cell; RBC, red blood cell; Neu; neutrophile; Lym, lymphocyte; Eos, eosinophils;Bas, basophils; Mono, monocytes; PLT, plaque level test; Hb, hemoglobin; ALT, alanine aminotransferase; AST, aspertate aminotransferase; CK, Creatin-Kinase; GGT, gamma-glutamyl transpeptidase; HBDH, hydroxybutyrate dehydrogenase; LDH, lactic dehydrogenase; Cl, chlorine; K, potassium; Na, sodium; P, phosphorus; PTA, prothrombin active; PT, prothrombin time; APTT, activated partial thromboplastin time; PTR, prothrombin time ratio; TT, thrombin time; CRP, C-reactive protein; PCT, procalcitonin; KL-6, Krebs von den Lungen-6; IgM, Immunglobulin M; IgG, Immunglobulin G; IgA, Immunglobulin A*.

### Correlation Between Antibodies and Clinical Outcomes and Laboratory Parameters

SARS-CoV-2-specific IgM, IgG, and IgA were significantly positively correlated with days of hospitalization (*P* = 0.023, *P* = 0.015, and *P* < 0.001, respectively) and days of PCR turning negative (*P* = 0.001, *P* < 0.001 and *P* = 0.001, respectively). The three specific antibodies were correlated with most laboratory parameters. A significant correlation was observed with specific IgM and *Eos (%)* (r = −0.162, *P* = 0.002), *hemoglobin (Hb)* (r = 0.113, *P* = 0.031), *alanine aminotransferase* (ALT) (r = 0.221, *P* < 0.001), *AST* (r = 0.155, *P* = 0.003), *glucose* (r = 0.117, *P* = 0.026), *gamma-glutamyl transpeptidase* (GGT) (r = 0.151, *P* = 0.004), *HBDH* (r = 0.124, *P* = 0.027), LDH (r = 0.158, *P* = 0.005), and *KL-6* (r = 0.190, *P* < 0.001). A significant correlation was observed between SARS-CoV-2- specific IgG and *albumin* (r = −0.131, *P* = 0.012), *globulin* (r = 0.174, *P* = 0.001), *ALT* (r = 0.153, *P* = 0.003), *creatine kinase (CK)* (r = −0.138, *P* = 0.014), *glucose* (r = 0.279, *P* < 0.001), *GGT* (r = 0.163, *P* = 0.002), *HBDH* (r = 0.193, *P* = 0.001), LDH (r = 0.216, *P* < 0.001), *D-dimer* (r = 0.024, *P* < 0.001), and *KL-6* (r = 0.405, *P* < 0.001). A significant correlation was observed between SARS-CoV-2-specific IgA and almost all laboratory parameters (*P* < 0.05), except for *Hb* and *CK* (Table 3).

**Table 3:** The relationships of levels of SARS-COV-2 specific antibodies with clinical outcome and laboratory parameters.

	**IgM**		**IgG**		**IgA**	
	**Coefficient**	***P***	**Coefficient**	***P***	**Coefficient**	***P***
**Clinical outcome**						
Days of hospitalization	0.120	**0.023**	0.127	**0.015**	0.234	**<0.001**
Days of PCR turning negative	0.353	**0.001**	0.408	**<0.001**	0.340	**0.001**
**Laboratory parameters**						
Eos (%)	−0.162	**0.002**	0.011	0.841	−0.136	**0.010**
Lym (%)	−0.037	0.478	−0.021	0.688	−0.109	**0.038**
Mono (%)	0.060	0.250	0.073	0.164	0.143	**0.006**
Hb	0.113	**0.031**	0.027	0.602	0.062	0.241
Albumin	0.053	0.953	−0.131	**0.012**	−0.234	**<0.001**
Globulin	0.081	0.122	0.174	**0.001**	0.177	**0.001**
ALT	0.221	**<0.001**	0.153	**0.003**	0.184	**<0.001**
AST	0.155	**0.003**	0.082	0.118	0.188	**<0.001**
CK	−0.045	0.427	−0.138	**0.014**	−0.052	0.358
Glucose	0.117	**0.026**	0.279	**<0.001**	0.239	**<0.001**
GGT	0.151	**0.004**	0.163	**0.002**	0.214	**<0.001**
HBDH	0.124	**0.027**	0.193	**0.001**	0.228	**<0.001**
LDH	0.158	**0.005**	0.216	**<0.001**	0.250	**<0.001**
D-dimer	0.054	0.301	0.204	**<0.001**	0.217	**<0.001**
CRP	0.004	0.938	0.055	0.313	0.211	**<0.001**
PCT	0.010	0.859	0.073	0.181	0.169	**0.002**
CD4+T%	−0.147	0.049	−0.115	0.123	−0.217	**0.003**
CD4+T/CD8+T ratio	−0.139	0.064	−0.059	0.430	−0.200	**0.007**
KL-6	0.190	**<0.001**	0.405	**<0.001**	0.364	**<0.001**
IgM	–	–	0.608	**<0.001**	0.586	**<0.001**
IgG	–	–	–	–	0.664	**<0.001**
IgA	–	–	–	–	–	–

*The results with P > 0.05 were not shown. P < 0.05 are shown in bold*.

*SARS-COV-2, severe acute respiratory syndrome coronavirus-2; IgM, Immunglobulin M; IgG, Immunglobulin G; IgA, Immunglobulin A; Lym, lymphocyte; Eos, eosinophils; Mono, monocytes; Hb, hemoglobin; ALT, alanine aminotransferase; AST, aspertate aminotransferase; CK, Creatin-Kinase; GGT, gamma-glutamyl transpeptidase; HBDH, hydroxybutyrate dehydrogenase; LDH, lactic dehydrogenase; CRP, C-reactive protein; PCT, procalcitonin; KL-6, Krebs von den Lungen-6*.

### Clinical Outcomes in the Three Groups and Antibodies With Different Severities

**Figure 1:**
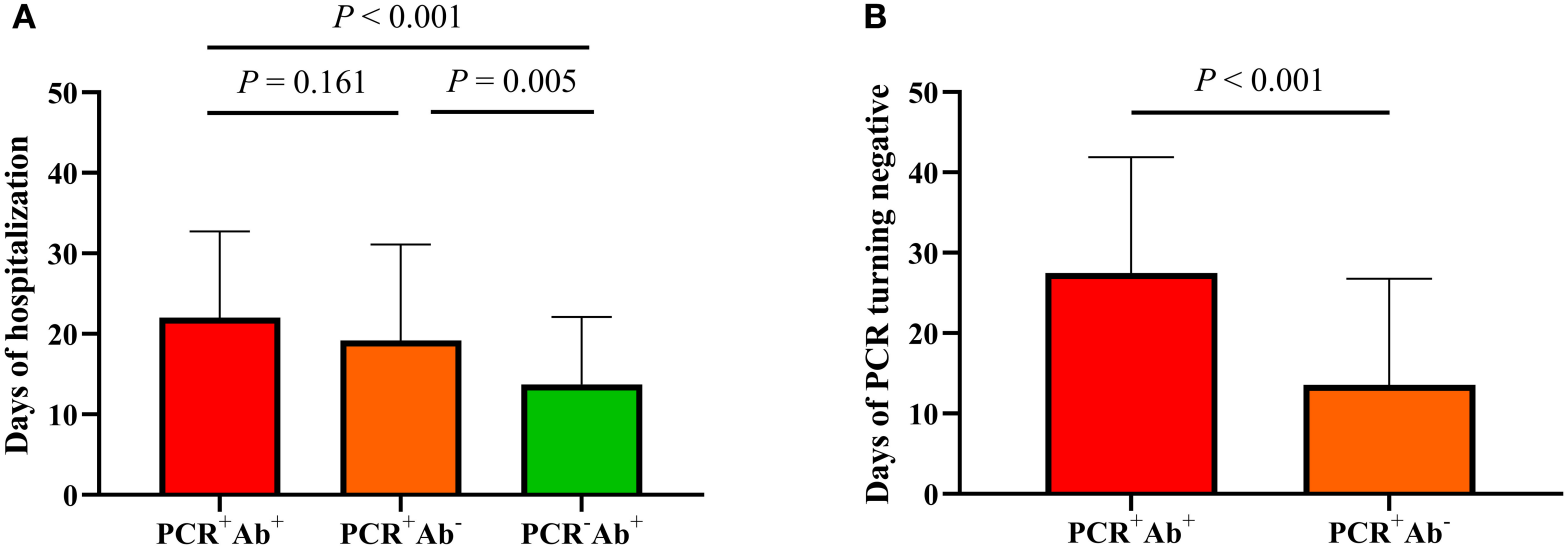
Comparison of clinical outcomes in COVID-19 patients grouped by the results of PCR and serological antibodies. **(A)** Days of hospitalization among three groups. **(B)** Days of PCR turning negative between PCR+Ab+ and PCR+Ab group.

**Figure 2:**
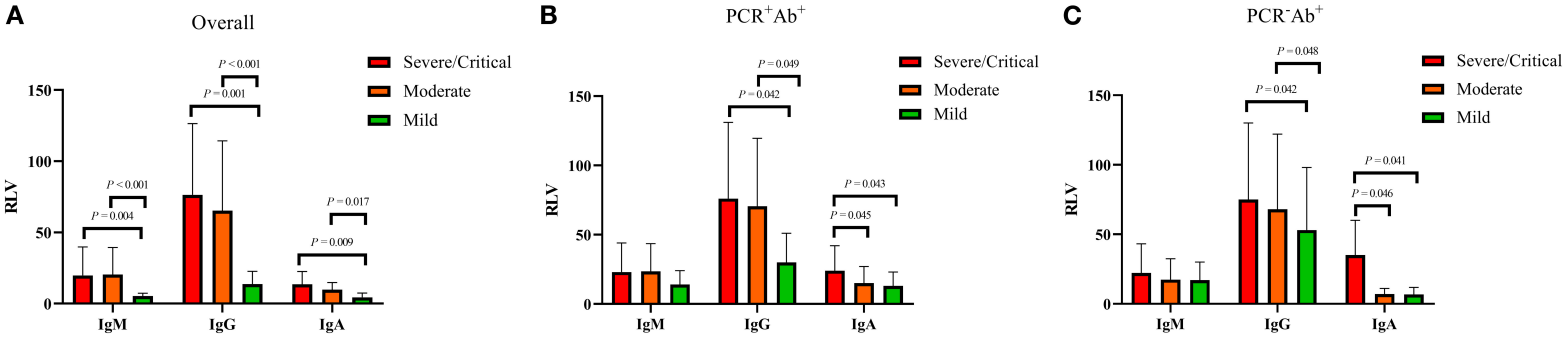
Comparison of antibody levels in COVID-19 patients with different severity. RLV, relative luminescence value. **(A)** Antibody levels in overall COVID-19 patients with different severity. **(B)** Antibody levels in PCR+Ab+ group with different severity. **(C)** Antibody levels in PCR-Ab+ group with different severity.

Among the three groups, patients in the PCR^+^Ab^+^ group had significantly longer duration of hospitalization than those in the PCR^+^Ab^−^ and PCR^−^Ab^+^ groups (both *P* < 0.001). However, there were no significant differences between the PCR^+^Ab^−^ and PCR^−^Ab^+^ groups (*P* = 0.085). Patients in the PCR^+^Ab^+^ group had significantly longer time for PCR turning negative than those in the PCR^+^Ab^−^ group (*P* < 0.001) (Figure 1). In the COVID-19 patients with different severities, the levels of SARS-CoV-2-specific IgM, IgG, and IgA were significantly higher in patients with severe/critical and moderate disease than in those with mild disease (*P* = 0.004 and *P* < 0.001, *P* = 0.001 and *P* < 0.001, *P* = 0.009 and *P* = 0.017, respectively). However, there was no statistically significant difference in specific antibodies between those with severe/critical and moderate disease (*P* > 0.05; Figure 2A). In the PCR^+^Ab^+^ and PCR^−^Ab^+^ groups, the levels of SARS-CoV-2-specific IgG and IgA were significantly higher in patients with severe/critical and moderate disease than in those with mild disease (*P* < 0.05), but specific IgM did not show significant differences between the two groups with different severity (*P* > 0.05) (Figures 2B,C).

### Network-Based Analysis of Clinical Outcomes and Laboratory Parameters

**Figure 3:**
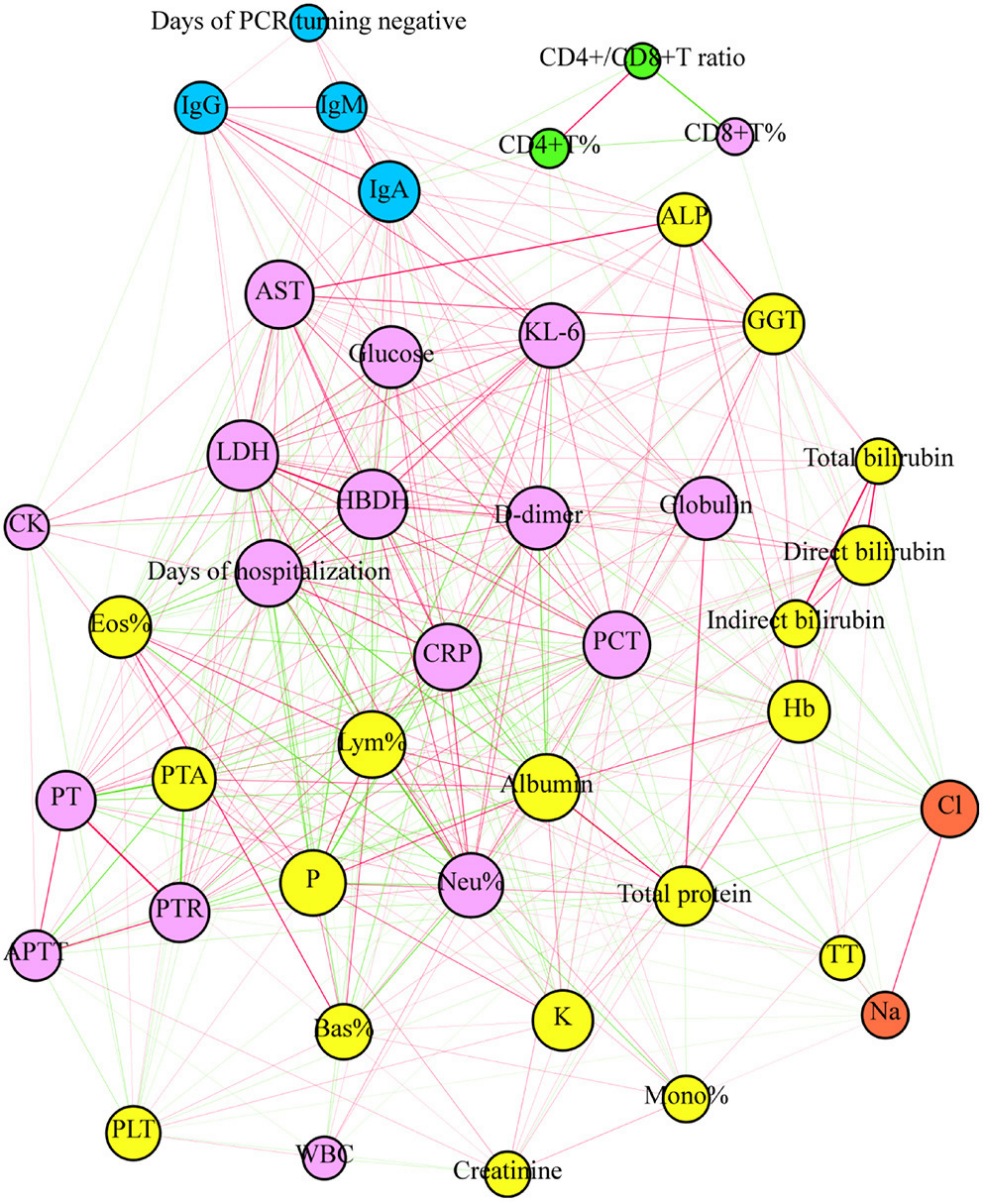
The plot of network analysis for the clinical outcome and laboratory parameters. The days of hospitalization and PCT turning negative and laboratory parameters were represented in the graph by a specific node who's color represents a specific cluster. The size of node is proportional to the sum of the edges that connect to them. Edges between nodes represent a statistically significant association (*P* < 0.05). The edge's thickness represents the strengths of their association (correlation coefficient, Spearman's correlation). The correlation coefficient is represented by color, with green and red indicating negative and positive relationships, respectively.

We performed a network-based analysis to further understand the network association of days of hospitalization and days of PCR turning negative with laboratory parameters (Figure 3). The network graph comprised 43 nodes, with each one representing clinical outcomes or laboratory parameters and a total of 418 links representing those correlations with a *P* < 0.05. The network graph comprised five clusters of highly interlinked nodes. Cluster 1 comprised 17 nodes (purple) which included 1 clinical outcome (days of hospitalization) and 16 laboratory parameters, including *AST, Kl-6, glucose, LDH, HBDH, D-dimer, globulin, PCT, CRP, CK, PT, PTR, activated partial thromboplastin time (APTT), Neu, WBC, and CD8*^+^*T%*. Cluster 2 comprised 4 nodes (light blue) which included 1 clinical outcome (days of PCR turning negative) and 3 antibodies (IgM, IgG, and IgA). Clusters 3 (yellow), 4 (green), and 5 (orange) included 18, 2, and 2 laboratory parameters, respectively.

### Severe Acute Respiratory Syndrome Coronavirus 2-Specific Antibodies to Predict Days of Polymerase Chain Reaction Turning Negative

**Figure 4:**
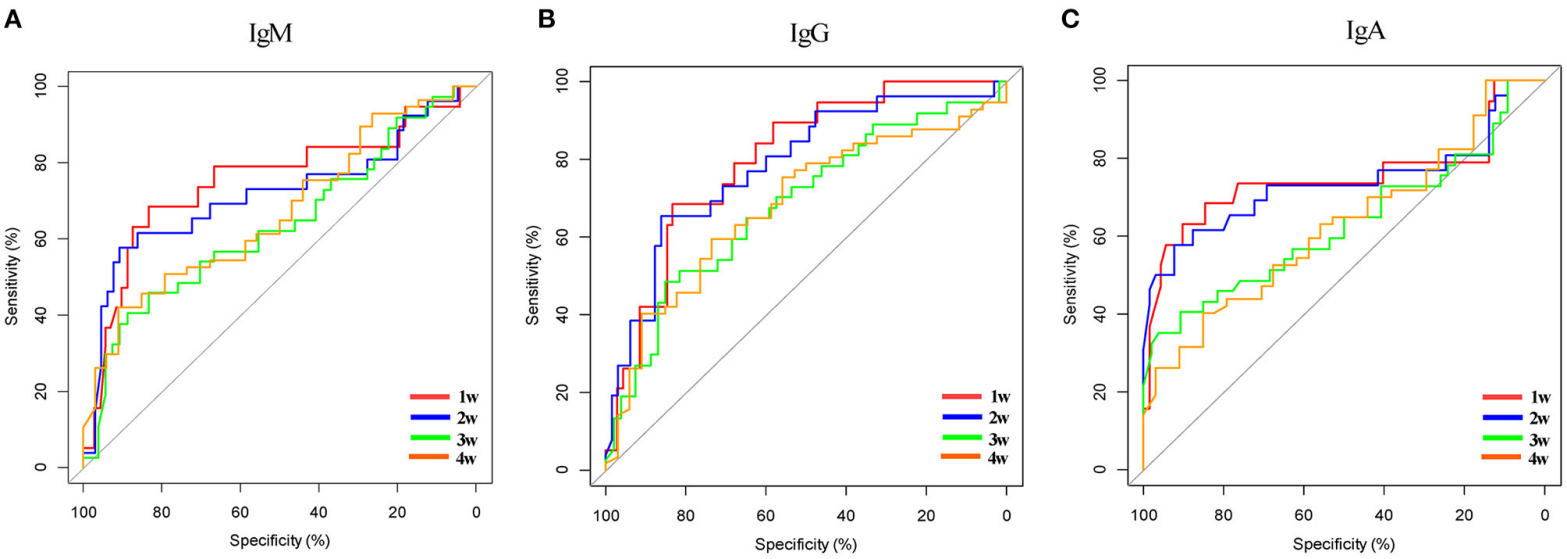
ROC curve for SARS-COV-2 specific antibodies to predict days of PCR turning negative in different time points. **(A)** Specific IgM to predict days of PCR turning negative in different time points. **(B)** Specific IgG to predict days of PCR turning negative in different time points. **(C)** Specific IgA to predict days of PCR turning negative in different time points.

**Figure 5:**
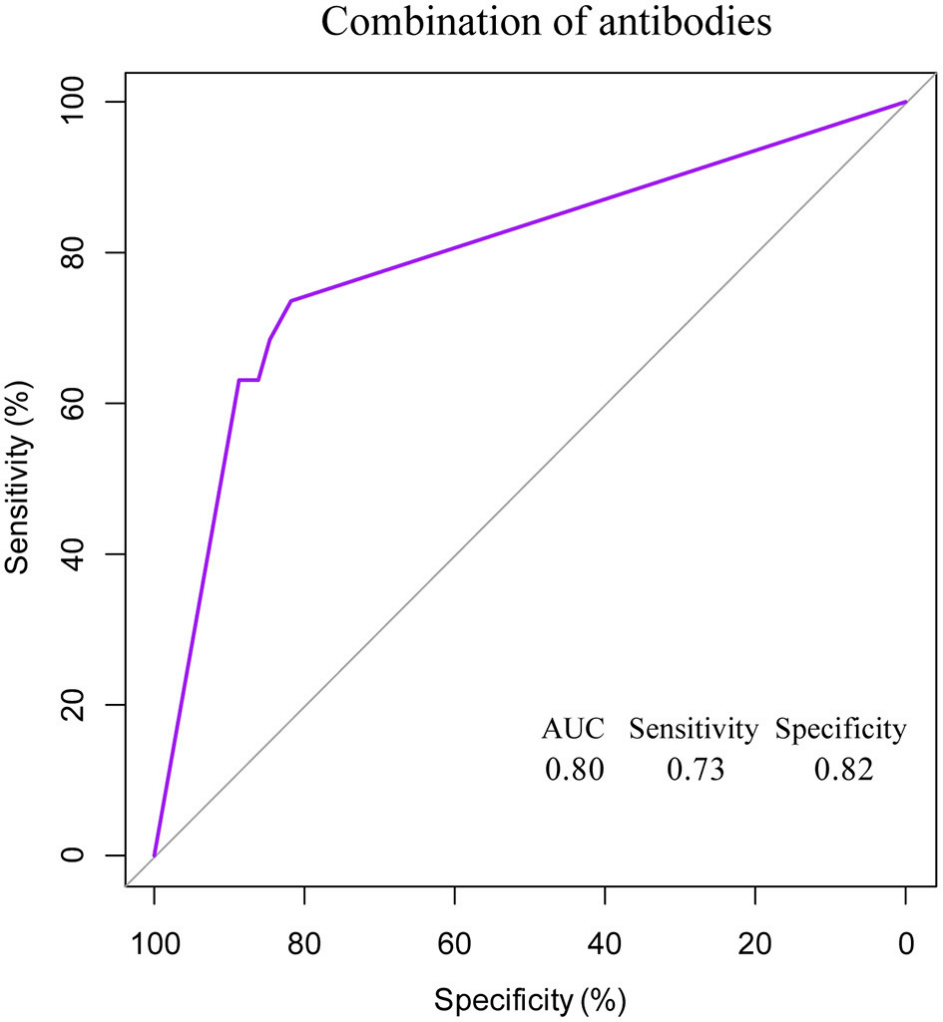
ROC curve for combined antibodies to predict PCR turning negative in 1 week.

In the network-based analysis, days of PCR turning negative was most strongly correlated with the three specific antibodies. Thus, we performed receiver operating characteristic (ROC) curve analysis for SARS-COV-2-specific antibodies to predict the days of PCR turning negative at different time points (Table 4, Figure 4). We found that the three antibodies were most effective in predicting the days of PCR turning negative within 1 week. When the cut-off values of IgG, IgM, and IgA was 3.2, 1.8, and 0.4, the sensitivity and specificity were 68 and 83%, 68 and 83%, and 63 and 90%, respectively. When the three antibodies were combined to predict the days of PCR turning negative within 1 week, we found that the best performance was achieved when the cut-off values of IgM, IgG and IgA were 3.2, 1.8, and 0.5, respectively, with a sensitivity of 73% and specificity of 82% (Figure 5).

**Table 4:** Summary of ROC curve analysis for SARS-COV-2 specific antibodies to predict PCR turning negative in different time points.

	**AUC**	**Cut-off**	**Sensitivity**	**Specificity**
**IgG**				
PCR turning negative in 1 week	0.80	3.20	0.68	0.83
PCR turning negative in 2 weeks	0.78	4.60	0.65	0.86
PCR turning negative in 3 weeks	0.68	4.60	0.48	0.85
PCR turning negative in 4 weeks	0.68	22.30	0.59	0.73
**IgM**				
PCR turning negative in 1 week	0.75	1.80	0.68	0.83
PCR turning negative in 2 weeks	0.72	0.60	0.58	0.91
PCR turning negative in 3 weeks	0.64	0.60	0.41	0.89
PCR turning negative in 4 weeks	0.67	2.00	0.42	0.91
**IgA**				
PCR turning negative in 1 week	0.75	0.40	0.63	0.90
PCR turning negative in 2 weeks	0.74	0.50	0.58	0.92
PCR turning negative in 3 weeks	0.63	0.20	0.35	0.96
PCR turning negative in 4 weeks	0.63	2.10	0.40	0.85

## Discussion

Based on our results, COVID-19 patients with PCR^+^Ab^+^ presented worse clinical features, laboratory abnormalities, and clinical outcomes, including longer days of hospitalization and more number of days of PCR turning negative, compared to those patients in the PCR^+^Ab^−^ or PCR^−^ Ab^+^ groups. In addition, the levels of IgG, IgM, and IgA were all significantly correlated with the days of hospitalization and days of PCR turning negative, along with having multiple correlations with other laboratory parameters. The network-based analysis suggested that the number of days of PCR turning negative was only strongly associated with the concentrations of IgM, IgG, and IgA, whereas the duration of hospitalization was closely associated with inflammatory and coagulation abnormalities. We further found that the levels of IgM, IgG, and IgA presented a favorable prediction for the days of PCR turning negative at 1 week after admission. In summary, our study provided evidence showing that patients with PCR^+^Ab^+^ tended to exhibit worse clinical characteristics and laboratory parameters, and subsequently worse clinical outcomes, compared to those with either PCR^+^Ab^−^ or PCR^−^Ab^+^. Furthermore, the levels of IgM, IgG, and IgA showed significant clinical relevance, and the value of these in disease management should be further investigated.

Fever, cough, dyspnea, and fatigue are the main symptoms of COVID-19 (Huang C. et al., 2020). We found that patients with PCR^+^Ab^+^ had a significantly higher incidence of major symptoms than patients with either PCR^+^Ab^−^ or PCR^−^Ab^+^. Furthermore, comorbidities, including hypertension, diabetes, and cardiovascular disease, which are risk factors for COVID-19 progression and death (Guan et al., 2020), were more prevalent in patients with PCR^+^Ab^+^ than in patients with PCR^+^Ab^−^ and PCR^−^Ab^+^. Currently, patient stratification is mainly based on disease severity and mortality. Previous studies have investigated laboratory abnormalities in the cohort of COVID-19 patients with severe disease and high risk of mortality and have identified several parameters that might serve as potential indicators of disease progression, including inflammatory, hematological, coagulation, and immune parameters (Chen et al., 2020; Liu S. et al., 2020; Wendel et al., 2020). In our study, patients with PCR^+^Ab^+^ showed more distinct panel of laboratory abnormalities compared with the other two groups, particularly presenting lower *Eos, Lym, Bas* counts and *total protein* levels, and higher *Neu* counts*, D-dimer, PCT, CRP* levels. Furthermore, the days of hospitalization and the days of PCR turning negative were significantly higher in patients with PCR^+^Ab^+^ than those in patients in the other groups. Our study, stratifying patients based on the results of PCR and Ab showed that patients with PCR^+^Ab^+^ exhibited more symptoms, comorbidities, laboratory abnormalities, and worse clinical outcomes. A possible explanation for this might be related to the simultaneous positive results of PCR, with Ab served as the indicator representing the peak period of immune response to the virus invasion. One modeling study showed that the sensitivity of PCR was 33% 4 days after exposure, while it was 62% on the day of symptom onset (Kucirka et al., 2020; Sethuraman et al., 2020; Wang W. et al., 2020). COVID-19 patients who recovered tended to present negative PCR results. Additionally, the increased antibody titer was suggested to be related to the increased viral/antigen load during SARS-CoV-2 infection (Liu et al., 2020b). Liu et al. (2020b) showed that patients in the ICU had higher antibody response compared with those not in the ICU. Studies have shown that COVID-19 patients have the highest viral load during symptom onset, which contributes to the rapid production of antibodies and enhances macrophage-mediated acute lung injury (Liu et al., 2019; To et al., 2020). It can be inferred that the higher the patient's viral load and antibody level, the more serious the symptoms will be.

PCR and serological antibody tests are the two main diagnostic methods for COVID-19, whose positive rate could be affected by both time of virus exposure and intensity of the immune defense response (Li and Ren, 2020). By controlling our patients who were hospitalized after 1 week since symptom onset and performing multiple PCR tests, the difference in the positive rate of PCR and serological antibodies test among patients with COVID-19 can be hypothesized to be predominantly affected by the virus clearance and antibody production by the immune system that is associated with disease severity. COVID-19 patients with severe disease had more prolonged viral shedding in a variety of tissues than did patients with mild disease (Wang Y. et al., 2020). However, in our study, the same antibody-positive patients did not have more severe disease than the nucleic acid-negative patients, and this may require further studies with larger samples.

A single-center retrospective study showed that (Liu X. et al., 2020) COVID-19 patients with severe/critical had a higher risk of clinical adverse events when the IgM titer was higher than 50 AU/ml, and a lower IgM titer in severe/critical patients may indicate a better prognosis. Hou et al. found that (Hou et al., 2020) IgM levels in COVID-19 patients with severe and critical disease were higher than those in patients with mild disease, while IgG levels in patients with critical disease were lower than those in patients with mild and severe disease. Zhang B. et al. (2020) found that patients with high IgG levels had more severe symptoms than those with low IgG levels. In the current study, we observed that the levels of antibodies were significantly higher in moderate and severe patients than those with mild disease. After excluding patients with negative results of antibody test, there was no difference in IgM between different severities. The findings were in line with our previous data (Huang Z. et al., 2020), which demonstrated that the IgG and IgA levels tended to be related to the disease severity. Meanwhile, we failed to found any difference in antibody levels between severe and moderate disease in the current study. However, the patients' antibodies levels could be affected by both viral load and the intensity of immune response within 1–2 weeks after onset of symptoms (Kwon et al., 2020). Although severe patients did not present a higher levels of antibodies on admission as compared those with moderate disease, it could not conclude that the levels of antibodies would not be upward with the disease progress in patients with severe disease.

In addition to providing qualitative results to diagnose COVID-19, the clinical relevance of antibody titer is limited. Therefore, we performed correlation analysis and found that the levels of antibody are associated with a variety of clinical laboratory parameters, including *Eos, Lym* and its subset, *PCT*, and *CRP*, which indicates that the production of antibodies might be involved in the immune response against COVID-19, strongly interacting with hematological, inflammatory, and coagulation systems. Indeed, hematological, inflammatory, and coagulation abnormalities contribute to disease severity (Ghweil et al., 2020; Tjendra et al., 2020; Zhang H. et al., 2020). We also found that IgA was significantly related to most blood test indicators, further indicating the importance of specific IgA. Our previous study also showed that (Huang Z. et al., 2020) IgA detection was more suitable in the early stages than IgM and has important reference value in the later stages of COVID-19. Currently, detection of SARS-CoV-2-specific antibodies is mainly focused on IgM and IgG; thus, paying sufficient attention to IgA is urgently required. Intriguingly, increased levels of IgM, IgG, and IgA were all correlated with longer days of hospitalization and days of PCR turning negative. Our data suggest that antibody detection might have additional clinical value beyond merely serving as a diagnostic tool. Considering that the clinical outcomes could also be related to other clinical parameters, we performed a network-based analysis comprising all clinical parameters and the two investigated clinical outcomes. Our results further confirmed that the levels of IgG, IgM, and IgA were only strongly associated with the days of PCR turning negative, while the days of hospitalization was closely associated with other clinical parameters, such as *PCT* and *CRP* levels. One potential explanation is that increased antibody levels on admission might be a sign of enhanced immune response at the early stage against COVID-19, which is significantly harmful to virus clearance and extends the days of PCR turning negative.

To further evaluate the clinical value of antibody levels, we performed ROC analysis to explore whether these antibodies could provide a favorable prediction of days of PCR turning negative. Nucleic acid negative result is the most important standard for patients to be discharged from hospital, and it is also an indicator to evaluate whether patients have infectivity. Liu et al. (2020a) found that the positive rate of nucleic acid results was above 60% in the first 11 days after symptom onset and then decreased rapidly; thus, 11 days after symptom onset, the diagnosis of SRAS-CoV-2 infection should mainly depend on the level of specific antibodies. However, our recommendation is that antibody and nucleic acid detection be performed simultaneously. Antibody detection can not only diagnose SARS-CoV-2 infection but also predict the time when nucleic acid result turns negative. We found that the single or combined IgM, IgG, and IgA were the most effective in predicting the negative conversion of nucleic acid within 1 week. This suggests that in the process of clinical diagnosis and treatment, patients with rapid negative nucleic acid conversion can be predicted and screened according to the antibody level. Different results of combined nucleic acids and antibodies predicted different clinical outcomes. We found that patients who were positive for both nucleic acids and antibodies had significantly longer hospital stays than patients who were positive for either and that antibody-positive patients had significantly longer nucleic acid conversion than antibody-negative patients. Thus, determining the results of both nucleic acids and antibodies allows us to assess the prognosis of COVID-19 to some extent.

For the first time, our study provides clinicians with a convenient method for predicting the length of hospital stay and time to nucleic acid conversion based on nucleic acid and antibody results on admission, but there are some limitations to this study. First, as a cross-sectional study, there was no dynamic observation of severity, laboratory parameters, or antibody levels, which may have influenced the final outcome. Second, the majority of patients had moderate disease, and there were significantly few patients with mild and severe/critical disease, which may have led to a bias in the results. Third, there may be a lag between time to nucleic acid turning negative and length of hospital stay due to a number of factors. For example, the time when a nucleic acid tests positive is not necessarily the time when a nucleic acid appears positive, and the patient may refuse to leave the hospital due to psychological factors. However, as a real-world study, we are convinced that our results are important for the clinical evaluation of COVID-19.

In conclusion, COVID-19 patients who were both positive for nucleic acids and antibodies had more severe symptoms, and longer hospital stays and longer time to nucleic acid conversion. The levels of the three antibodies were highest in severe/critical cases and lowest in mild cases, but there was no difference in antibody levels between patients with moderate and severe/critical disease. Three specific antibodies were positively correlated with days of hospitalization and time for nucleic acid test turning negative, and were predictive of time to nucleic acid conversion. Of the three antibodies, IgA is associated with more laboratory parameters, suggesting that IgA testing is also important and should not be overlooked in the diagnosis of COVID-19.

## Data Availability Statement

The raw data supporting the conclusions of this article will be made available by the authors, without undue reservation.

## Ethics Statement

This study was approved by the Ethics Committee of Wuhan Central Hospital Medical (Research Ethics No. 1, 2020). Considering the infectivity of COVID-19, informed consent was not required to be signed.

## Author Contributions

BS, HW, and HC conceived and designed the project. HC and HW performed the experiments. HC and RQ analyzed the data and wrote and revised the manuscript. ZH, LH, WL, PZ, and HH mainly collected the clinical data. All authors contributed to the article and approved the submitted version.

## Conflict of Interest

The authors declare that the research was conducted in the absence of any commercial or financial rel that could be construed as a potential conflict of interest.
